# Nuts and Bolts of a POCUN Program

**DOI:** 10.24908/pocus.v7iKidney.15346

**Published:** 2022-02-01

**Authors:** Noelle M Northcutt, Nathaniel C Reisinger

**Affiliations:** 1 Division of Hospital Medicine, Denver Health Hospital; 2 Penn Medicine, Penn Kidney

**Keywords:** nephrology, POCUS, POCUN, NephroPOCUS, program development

## NUTS and BOLTS of a POCUN Program

For those that have experienced how much point of care ultrasound can positively impact patient care, the potential of an organized point of care ultrasound program is moderated by the reality of building such a program from scratch. We have watched novice and intermediate users ride the roller coaster of ambition and reality checks at each of the hands-on skills sessions across the nation. The first climb occurs on the morning of Day 1. Anticipation grows over how hard it might be to learn this skill, then peaks and dips into the exhilaration of seeing success with what feels like minimal guidance. A comprehension of attainability takes over, and after each hands-on session, confidence climbs fast. Day 2 begins with the courage of some experience, and the anticipation of more success. The questions start as the climb up the second hill begins: “This is practice changing! How do I start a program at my home institution?” The ride turns into a steady flow of dips, spins, and twists as facilitators use less guidance and allow for more struggle. The questions turn to statements as learners develop a metered sense of capability: “I NEED a program at my home institution. My colleagues are going to want to know how to do this too, and I want to develop my skills.” 

The momentum is strong for physicians to integrate Point of Care Ultrasonography (POCUS) across the spectrum of ambulatory and inpatient care. Nephrologists can interpret and evaluate patients using point of care ultrasound in nephrology (POCUN) and it is becoming a key part of training 1. The promise of POCUS for improved patient care is supported steadily as studies demonstrate how it can expedite more accurate diagnoses and improve outcomes 2, 3. Indeed, POCUN may be a key component of restoring interest in nephrology with ultrasound interpretation the second most desired topic for additional instruction 4. So how does one get from attending a professional conference to something organizationally developed enough to be called a program in order to realize these benefits? Here are some basic questions to ask yourself (or your team!) to mature a POCUS learning experience into a structurally sound program.

## BUILDING

### Who Are Your Patients?

Making the biggest possible impact with POCUS requires an alignment of the POCUS user’s skillset with the patient’s needs. Patients with kidney disease use acute care services disproportionately and POCUS can decrease the burden of care experienced by a patient at a reduced cost to the system as a whole 5. As a sub-specialist, a program’s scope can be narrowed and thus the impact concentrated. Asking “Who are your patients?” is really saying: “Define your core POCUS applications.” POCUS puts the power of ultrasound into the hands of the clinician already at the bedside 6. Bedside physicians are hungry to learn techniques that improve their ability to diagnose patients—especially toward the elusive accurate volume assessment 7. In the hands of a nephrologist, the addition of lung ultrasound alone to the POCUN toolkit can assist in assessments of lung water which can improve intradialytic ambulatory blood pressure control and changes in pulmonary edema in response to dialysis 8, 9, 10. To complement lung POCUS, diagnostic assessment of the IVC, kidneys and bladder, and focused echo are limited POCUS exam additions that support a more accurate volume assessment. Ultrasonographic findings in COVID-19 may complicate the interpretation of lung ultrasound for volume assessments in particular 11. Even as it appears the COVID-19 pandemic is waning, the experience exemplifies how emerging diseases can impact our diagnostic tools’ limitations.

Visualization of common kidney and bladder pathologies such as hydronephrosis, cysts, stones, prostatomegaly, and masses are possible with a basic POCUN skill set. Basic echocardiography can evaluate for pericardial effusion, gross valvular abnormalities, and gross assessments of ejection fraction. Assessment for maturation of the arteriovenous fistula and guidance of vascular access cannulation are also potential adjuncts to key procedural ultrasound skills for central venous catheter placement and renal biopsy 12, 13.

### Who is Your Team?

Knowing who to recruit will help expedite organization and communication at the early stages of program design and eliminate obstacles during implementation and execution phases. The cornerstone of a program, of course, is the faculty that can use POCUS clinically and become part of the fabric of the program. Beyond capable physician faculty within the nephrology division that will perform the scans, relationships with others outside nephrology are critical to program success. Non-physician sonographers are part of those teams for many institutions, for example, and can be an outstanding asset for one-on-one hands-on instruction of learners. Having an established relationship with radiologists can lead to a robust image review and quality assurance team. Cardiologists are an excellent addition for focused echocardiographic reviews. Emergency medicine physicians benefit from decades of experience with POCUS implementation and education. Departmental collaboration increases the educator pool for all departments. Collaboration can also streamline impact on patient care. For example, a multi-department collaborative can perform as a committee when implementing standardized documentation of POCUS findings which would allow tracking of findings over time for an individual patient. Information Technology (IT) is crucial in developing an efficient and seamless way to store images for both review and billing purposes, not to mention integration into security frameworks so that personal health information (PHI) remains protected. Many of the newer handheld devices utilize wireless information uploading and cloud-based image storage. Successful image storage integration into existing structures such as picture archive communication system (PACS) is crucial. The biomedical and engineering teams also have a stake in equipment to be purchased by a hospital and used by faculty in the care of patients, especially at the inevitable point of needing repair or replacement. The support of your executive leadership can make for more fluid program-building as the development of a program from scratch inevitably requires investment for equipment and IT workflow solutions, even if the plan is simply to integrate into an existing electronic medical record and Digital Imaging and Communications in Medicine (DICOM) PACS storage solution.

### Best Tools for the Job

If you are talking about clinical POCUS, then the choice is really between a handheld device and a small cart-based ultrasound. There are now multiple options in both categories making the devices competitive in class. **Table 1 **provides a quick rundown of pros and cons for each. One place where they differ greatly is in price comparisons. Handhelds are increasingly subscription and cloud based. This means a pay structure with either yearly or monthly fees in addition to hardware costs in order to maintain access to image storing clouds and/or device tools for interpretation and education. In contrast, cart or laptop-based systems are currently still offered as one-time hardware costs with a more traditional warranty structure. The outright cost for handhelds ranges from $2000 to $8000 with subscriptions costing an additional amount yearly depending on subscription type (personal versus group license) 14, 15. Cart and laptop systems typically used in bedside applications range from $25,000 to $60,000 16. Program goals may change over time and with expansion so device demand can be expected to evolve. A new program for a nephrology practice with fellows may choose handhelds for inpatient and outpatient flexibility of using a mobile platform. An outpatient practice may choose to invest in a cart-based machine for a device that can provide excellent visualization for both procedural and non-procedural POCUS applications. One key feature setting apart larger and more expensive systems is pulsed-wave Doppler. Pulsed-wave Doppler is not required to perform core applications including lung, kidney, and bladder ultrasound, but can add information to advanced cardiac and renal vascular techniques.

**Table 1:** Table 1. Quick Pros and Cons of Handheld and Cart or Laptop POCUS Devices

**Handheld Pros**	**Handheld Cons**	**Cart/Laptop Pros**	**Cart/Laptop Cons**
Handheld / pocket sized	Potential for displacement/loss/ difficult to track	Better image quality due to better transducer quality	Less portable / maneuverable across multiple units
Can limit transducer style (and cost) based on focused applications	Limited applications if only one type of transducer purchased	Larger screen and controlling buttons	Dexterity at bedside also potentially limited due to size
Most with easy connections to cell phone / app-based	May require additional screen / tablet negating benefits of portability	Potential for all transducers needed on hand	More expensive than handheld devices

### Communication Planning

Patients can have a more positive healthcare experience and a better understanding of their diagnoses and care when POCUS is utilized 17, 18. A program administration communication strategy is key, but the most critical communication plan is at the patient level. How will you set expectations regarding the experience of an ultrasound and describe using patient-centered language what the grayscale means? Improved bedside doctoring is one of the most attractive parts of POCUS and effectively engaging the patient takes a different effort compared to the current workflow, i.e. sending the patient to a different department and communicating findings via the phone or the electronic medical record 19. Having a high standard of communication helps achieve the value of bedside ultrasound. How will your team communicate abnormal findings in real time? How will your team communicate uncertainty and the potential need for additional or repeat testing based on your bedside findings? What implications do your bedside findings have? For instance, if in the outpatient visit you diagnose new large pleural effusions what is the pathway for care? What if you note a new kidney mass when you performed a study looking for hydronephrosis? Communicating abnormal or unexpected findings are already a part of every physician’s toolkit; however, a plan for this type of POCUN communication should be considered a fundamental part of a program build. This is especially important with the advent of legislation similar to Pennsylvania Act 112 mandating written communication of “significant abnormalities” detected on imaging 20. 

### Quality Assurance and Image Review

Quality assurance (QA) and image review are a fundamental part of any POCUS program fulfilling many key functions including creating a teaching file of pathologic images, providing feedback to learners, building a learner image portfolio for credentialing, and communicating missed or incidental findings to patients. Setting up your team to include POCUS clinicians from other divisions and departments can become valuable when organizing the approach to image review and QA. Image review requires a reliable, retrievable, and organized collection of images to be stored securely. How to accomplish this can be fairly individualized, but there is one aspect that is universal: person-power. No matter what, a person or a team will need to have the time allotted to perform basic image review for quality assurance. When thinking about the image storage location, some of the most common solutions are a HIPAA compliant cloud, DICOM PACS, or third-party HIPAA compliant server. Transferring the images from the device to the storage solution will involve the IT team. Once uploaded, the POCUS clinical team will need a method to determine how images are selected for review. For instance, should all trainee-generated images be pushed to the patient’s chart or should poor quality images remain sequestered in the image archiving solution? The method for organizing QA solutions is individualized to suit the needs of the participating reviewers and the institutional demands. 

Some percentage of total images should be reviewed to ensure image acquisition and interpretation meets departmental thresholds for accuracy. Who participates in image review, how often it occurs, and what percentage of images are reviewed are part of the program design. Some programs will choose to QA 100% of images, whereas more established programs may only review 5-10% 21. Generally speaking, the more novice the user, the more images should be reviewed per term. Therefore, any program design should consider the number of novice users when allotting time for this effort. An academic nephrology fellowship program may choose to integrate a tiered approach to image review. For example, the POCUS faculty would oversee a review session where the fellow performs primary image review of images collected by the entire program with guidance. In this way the fellow develops the skill of reviewing with oversight. 

Establishing criteria for what constitutes a clinically adequate image supports users in performing clinically adequate scans as well as a structure for critique and improvement. The minimum of “pass/fail” image review does little to support the user in neither improving nor troubleshooting. Providing written or verbal feedback better supports the user and ultimately the patient.

A robust system for QA lays the groundwork for continuous quality improvement (QI). QI in POCUS is the process of evaluating the system as a whole and systematically investigating and implementing specific measures for the furtherance of patient care and trainee education using bedside ultrasound.

### Maintenance of Skills: Credentialing, Certification, and Privileging

Kim, Thiessen, and Strony break down the differences between these terms as specifically applied to POCUS 22. The residency-trained POCUS provider takes a different pathway to achieve competency compared to a practicing physician adding to their skills through a practice-based pathway. Whether credentialing is best for your new program is fully dependent on the existing structures present to ensure all physicians are qualified to practice at the level at which they are employed. Proponents of a requirement for credentialing and privileging emphasize the diversity of training pathways and the lack of POCUS milestone requirements from neither the ACGME for non-emergency medicine trained physicians nor most residencies. Without longitudinal oversight and milestone achievement as is expected for other skills such as taking a history and performing a physical exam with a stethoscope, the concern is that there could be excessive variability in competency, especially when requiring a physician to self-referee 23. Alternatively, credentialing and privileging requirements could be prohibitive to pursuing training in POCUS, as it raises a barrier based on an assertion that physicians could not be expected to effectively self-referee for a non-invasive diagnostic skill. In fact, the very fact that it is non-invasive is often held up as analogous to the use of the stethoscope, a skill in which no physician is required to demonstrate ongoing competency beyond their training years and for which one is not required to request special privileges 24.

Whether or not a system requests credentialing as a pathway to obtaining privileges, or permission to use POCUS is a reflection of the unique medical structure at each practice site. Certification programs can serve as surrogate practice-based pathways when residency pathways or hospital-based pathways are unavailable, or even in addition to those pathways. While not required to demonstrate competency, certification in aspects of clinical ultrasound—as can be obtained through the American Society of Diagnostic and Interventional Nephrology—serve as a bolster for nephrology faculty specializing in POCUS, signaling to peers and institutions that they are committed and capable in the field 25. Ultimately, how a new program grapples with this grey area can be expected to be informed by the ongoing national conversations on these topics. As an increasing number of residency and fellowship programs incorporate POCUS training and associated milestones into their curricula, more specialties will be able to consider POCUS a core competency, thus potentially obviating the need for special privileges 26.

## PROGRAM GROWTH and MAINTENANCE 

### Role of the POCUN Director 

The aforementioned foundational structure is well-served by having a designated leader with clearly delineated responsibilities 21, 27. Even if a program plans to launch with a team of leaders, the benefit of having a single point-of-contact for more efficient communication between team members and the larger POCUS network at a site cannot be overstated. Just for the structure outlined thus far there is a need for cross-disciplinary collaborations, general program oversight, and a person to spearhead organization of QA/QI. 

At every branchpoint of growth there is a corresponding number of responsibilities. Growth and maintenance will not likely occur without a designated POCUN Director. As each part of a program expands, the administrative burden grows. Growth in education such as the addition of a fellowship and education at the student or resident level requires recruitment, marketing, and curricular design. Growth in number of clinical users adds to the number of studies for QA as well as the responsibilities for oversight. Growth in the number of devices corresponds to the number of times something needs a fix, repair, or simply guidance for use. The role and responsibilities of POCUN Director deserves protected time to be most effective.

### Fellowship

How does a program become self-sustaining? Expanding the core nephrology faculty is an obvious first goal in order to allow for shared responsibilities across the spectrum of administrative and educational need. There is no one best way to expand the faculty leadership cohort. Integrating POCUS faculty development into existing responsibilities is possible with approaches curated to the specialty with respect to demands on the participants time and in tandem with learners at different career stages 27, 28, 29. Efficiency in leadership tasks like image review and quality assurance programming can be expected to be dependent on supported time for a coordinating program director 30. 

Integrating POCUS into the core fellowship curriculum is one way to grow a foundational program into a sustainable cohort of skilled bedside sonographers and expand a programs capacity for image review. Koratala, et al. describe one such method of building POCUS education and skill development into the first 18 months of nephrology fellowship training 31. Offering a dedicated fellowship in POCUS is also an option for further expansion into educational POCUS training at every level of the medical career pathway which would be expected to translate into a more longitudinally trained cohort. As described by Koratala, et al., “As more medical students and residents are trained in POCUS, it is conceivable that the focus of POCUS training during fellowship will shift from ‘performing’ the examinations to ‘interpreting’ them, and the required training time will decrease.” Non-ACGME fellowships in POCUS require a department or departments to be able to budget for the expense of a fellow’s clinical salary and administrative costs for program director’s time in return for the expected benefit of an additional educator for the student and resident trainees and longitudinal growth of the program 32. 

### Documentation and Billing

Documentation to support billing includes the indication for the study, the type of exam, findings, and the note-writer’s interpretation of these findings as well as at least a still image, saved and retrievable 33. Applying appropriate ICD-10 and CPT codes for the POCUN studies you most often perform will benefit from working with your billing and coding experts to ensure the documentation will support billing. An in-depth review of documentation and billing is beyond the scope of “Nuts and Bolts” and recently published frameworks such as “Billing I-AIM” by Hughes, et al., provide an excellent and detailed discussion of POCUS billing that is generalizable to nephrology or other subspecialty practices 34. While investigation specific to nephrology is lacking, implementation of billing for POCUS activities in a single-center experience at a tertiary care emergency department produced a net profit within one year. Niyyar and O’Neill report billing for ultrasound at an academic center performed over 20,000 point of care studies in 20+ years 13. While it is an oft-cited worry that performing and billing for ultrasound will lead to increased liability, Niyyar and O’Neill noted no liability concerns in their time performing POCUS. This is substantiated by literature from perinatology and emergency medicine which failed to identify a single case of liability due to misdiagnosis that found no cases against internal medicine, family medicine, or pediatric physicians 35, 36. 

### What about education and competency?

Excluded from this review thus far has been any mention of a specific educational pathway for those looking to start a POCUN program or expand their ultrasound using cohort. A one-day course is enough to light the fire, but how does one keep it stoked and challenged? Hands-on courses, online content and curricula, and even Twitter are the modern-day core content providers 1. Robust online educational resources offer a compendium of how-to videos, literature summarized into easy-to-digest infographics, and pocket-guides for quick “just-in-time” review on a personal phone prior to scanning 37. Coupled with short-courses, workshops held by national conferences, and mini-fellowships, the online educational resources to support the launch of a new program already exist in volumes as free, open access medical education online material (FOAMEd) 38.

How to move POCUS users engaged in a program from curious to competent remains a black box even in 2021, as there is an ongoing lack of sufficient evidence on the best methods to assess competency 39, 40. While a handful of assessments exist for specific applications, how to deploy these to bridge learners between educational and clinical scanning is also not standardized 41. Until the evidence exists, this latitude shifts the burden of defining competency onto POCUS program directors and local expertise. The advantage to this is that it lends to a curated educational experience for the learners at a particular program. For example, some trainees may succeed in a volume-based approach (i.e., total number of scans to achieve a skill) while others succeed in a more objective skills-based approach (i.e. the skill is achieved when all boxes are checked in the pathway designed). The curricular design by the program director can be tailored to both.

### Starting a POCUS Program During a Pandemic

Considering a physician needs time, creative head space, and motivation to pursue developing a new program in their department, there are more than a few ways the current pandemic might stunt this type of growth. What is lost in the limitations to networking at national conferences or in-person courses is potentially a gain for the increased physical presence of the local cohort at one’s home institution. And the pandemic has shifted many national organizations to virtual formats for education with additional opportunities for online learning growing steadily. Troubleshooting the challenges of COVID-19 imposed physical distancing restrictions with the need for personal protective equipment (PPE) preservation is already underway by POCUS educators 42, 43. Ultimately the components of a well-designed program rely on local personnel to effectively collaborate in their commitment to accurate image acquisition and interpretation. Thankfully, collaboration amongst medical partners has prevailed during COVID-19, and sets a precedent for a future anticipated to include other emerging pathogens 44, 45. 

## Conclusion

These Nuts and Bolts can build a program in many different ways. There is no specific timeline to follow to engage potential teammates. A starting program in a site with three nephrologists well-trained in POCUN with strong administrative support and easy collaboration across departments would be expected to progress beyond the basics of program development rather quickly. On the other hand, a solo aficionado working to increase their skills through a national certificate program while simultaneously building the scaffolding for a well-designed program could be expected to need several years to accomplish the network described above. Invariably, there will be more that is unexpected than is planned, but that is the allure of building something from scratch. With a plan for each of the basic pieces described above, a new program will have solid footing on which to start.

## Disclosures

None.
